# Monitoring and evaluation of breast cancer screening programmes: selecting candidate performance indicators

**DOI:** 10.1186/s12885-020-07289-z

**Published:** 2020-08-24

**Authors:** Sergei Muratov, Carlos Canelo-Aybar, Jean-Eric Tarride, Pablo Alonso-Coello, Nadya Dimitrova, Bettina Borisch, Xavier Castells, Stephen W. Duffy, Patricia Fitzpatrick, Markus Follmann, Livia Giordano, Solveig Hofvind, Annette Lebeau, Cecily Quinn, Alberto Torresin, Claudia Vialli, Sabine Siesling, Antonio Ponti, Paolo Giorgi Rossi, Holger Schünemann, Lennarth Nyström, Mireille Broeders, On behalf of the ECIBC contributor group, Mariangela Autelitano, Edoardo Colzani, Jan Daneš, Axel Gräwingholt, Lydia Ioannidou-Mouzaka, Susan Knox, Miranda Langendam, Helen McGarrigle, Elsa Pérez Gómez, Ruben van Engen, Sue Warman, Kenneth Young, Cary van Landsveld-Verhoeven, Donata Lerda, Zuleika Saz-Parkinson, Elena Parmelli, Annett Janusch-Roi

**Affiliations:** 1 Department of Health Research Methods, Evidence, and Impact, Faculty of Health Sciences, McMaster University, Hamilton, Ontario Canada; 2 Iberoamerican Cochrane Center, Instituto de Investigación Biomédica Sant Pau (IIB Sant Pau-CIBERESP), Barcelona, Spain; 3 European Commission, Joint Research Centre, Via E. Fermi 2749 – TP 127, I-21027 Ispra, VA Italy; 4 Institute of Global Health, University of Geneva, Geneva, Switzerland; 5 IMIM (Hospital del Mar Medical Research Institute), Barcelona, Spain; 6 Queen Mary University of London, London, UK; 7 National Screening Service, Dublin, Ireland; 8 UCD School of Public Health, Physiotherapy & Sports Science, Dublin, Ireland; 9 German Cancer Society, Berlin, Germany; 10 CPO-Piedmont - AOU Città della Salute e della Scienza, Torino, Italy; 11 Cancer Registry of Norway, Oslo, Norway; 12 University Medical Center Hamburg-Eppendorf and Private Group Practice for Pathology, Hamburg, Germany; 13 St. Vincent’s University Hospital, Dublin, Ireland; 14 ASST Grande Ospedale Metropolitano, Milan, Italy; 15 Netherlands Comprehensive Cancer Organisation (IKNL), Utrecht, Netherlands; 16 University of Twente, Enschede, Netherlands; 17 AUSL Reggio Emilia, IRCCS, Reggio Emilia, Italy; 18 Department of Epidemiology and Global Health, Umeå University, Umeå, Sweden; 19 Radboud Institute of Health Sciences, Radboud University Medical Center, Nijmegen, Netherlands

**Keywords:** Breast neoplasms/diagnostic imaging*, Early detection of Cancer*/methods, Female, Mass screening/methods, Programme evaluation, Quality indicators, Health care/standards*

## Abstract

**Background:**

In the scope of the European Commission Initiative on Breast Cancer (ECIBC) the Monitoring and Evaluation (M&E) subgroup was tasked to identify breast cancer screening programme (BCSP) performance indicators, including their acceptable and desirable levels, which are associated with breast cancer (BC) mortality. This paper documents the methodology used for the indicator selection.

**Methods:**

The indicators were identified through a multi-stage process. First, a scoping review was conducted to identify existing performance indicators. Second, building on existing frameworks for making well-informed health care choices, a specific conceptual framework was developed to guide the indicator selection. Third, two group exercises including a rating and ranking survey were conducted for indicator selection using pre-determined criteria, such as: relevance, measurability, accurateness, ethics and understandability. The selected indicators were mapped onto a BC screening pathway developed by the M&E subgroup to illustrate the steps of BC screening common to all EU countries.

**Results:**

A total of 96 indicators were identified from an initial list of 1325 indicators. After removing redundant and irrelevant indicators and adding those missing, 39 candidate indicators underwent the rating and ranking exercise. Based on the results, the M&E subgroup selected 13 indicators: screening coverage, participation rate, recall rate, breast cancer detection rate, invasive breast cancer detection rate, cancers > 20 mm, cancers ≤10 mm, lymph node status, interval cancer rate, episode sensitivity, time interval between screening and first treatment, benign open surgical biopsy rate, and mastectomy rate.

**Conclusion:**

This systematic approach led to the identification of 13 BCSP candidate performance indicators to be further evaluated for their association with BC mortality.

## Background

Breast cancer (BC) remains a major public health issue in the European Union (EU) [1–3]. Currently, the vast majority of European countries operate population-based breast cancer screening programmes (BCSPs) [4]. However, the considerable variation in both incidence and mortality rates between European countries suggests inequalities in care among European citizens, including performance of the BCSPs [5].

Monitoring and evaluation of BCSPs is necessary to ensure that the programmes are as effective as expected. The basis for these activities is described in the European Guidelines for Quality Assurance in Breast Cancer Screening and Diagnosis [6, 7]. In general, a distinction should be made between 1) monitoring the performance of the screening programme via performance indicators that reflect the provision and quality of the activities constituting the screening processes and 2) evaluation of the impact of a screening programme as a whole based on the main outcomes. Although some evidence exists for both aspects [4, 7–10], the association between BCSP performance indicators and important patient outcomes, such as BC mortality, quality of life and undesirable effects, is poorly explored. If established, this would allow for more efficient monitoring and evaluation of the BCSPs. However, the few studies that have examined the association are limited in their methodologies, in the number of performance indicators evaluated, and report conflicting results [1–14].

In this context, the European Commission Initiative on Breast Cancer (ECIBC) aims to enhance the quality of BC care in Europe by developing a quality assurance (QA) scheme for the full spectrum of BC services [15, 16] and provides evidence-based guidelines for screening and diagnosis. The European Commission’s Joint Research Centre (JRC) is responsible for the overall scientific coordination and funding, also ensuring conflict of interest management, and transparent reporting of the activities. As part of the work, the Guidelines Development Group’s (GDG) subgroup on Monitoring and Evaluation (M&E) was tasked to identify potential BCSP performance indicators and their acceptable and desirable levels using a systematic and evidence-based approach. The main objective was to provide guidance on the use of BCSP performance indicators, monitoring of which would evaluate the effectiveness of breast cancer screening related to breast cancer mortality reduction a certain number of years after implementation. The purpose of this paper is to document the methodology used to identify candidate BCSP performance indicators, which will then be further evaluated for association between each one of them and BC mortality. The methodology for the latter will be described in another paper.

## Methods

The final list of potential performance indicators was identified through a multi-stage process: 1) conduct of a scoping review to identify a list of existing performance indicators; 2) development of a conceptual framework to inform indicator selection; 3) conduct of a survey among the M&E subgroup members to select a list of candidate performance indicators according to pre-agreed criteria; and 4) description of a BC screening and diagnostic pathway to facilitate the mapping of the indicators along the key steps. The process was guided by a pre-defined study protocol (unpublished) and completed in 2016–18. Representing various EU states, the M&E subgroup consists of European experts in breast cancer screening and diagnosis. To ensure synergy and consistency in the input of ECIBC working groups, two members of ECIBC’s Quality Assurance Scheme Development Group (QASDG) for the European quality assurance scheme in breast cancer services were included in the M&E subgroup as contributors. In addition, the selection of the candidate indicators was discussed at meetings of the full GDG and QASDG.

### Scoping review of performance indicators

First, a search in MEDLINE and EMBASE databases was conducted by Cochrane Iberoamerica to identify publications in English that report performance indicators in the context of BCSPs (Additional file 1). Editorials, debate articles, or conference abstracts were excluded. The key inclusion criterion was that the data must originate from population-based BCSPs implemented at regional or country level. After an initial calibration using a sample of the retrieved records, two reviewers each screened half of the study titles and abstracts for potential eligibility, according to the inclusion criterion. The reviewers then independently confirmed the eligibility based on the full text assessment. In case of discordance, consensus was reached by discussion or involving a third reviewer. A PRISMA flowchart was used to report the search flow [17].

Second, an extensive review of grey literature and expert consultation was carried out to identify performance indicators recommended and/or reported by BCSPs and national/regional authorities in charge of those programmes. Following consultations with M&E subgroup members, a sample of 12 countries (Australia, Canada, Denmark, Finland, Germany, Italy, Netherlands, New Zealand, Norway, Spain, Sweden, UK) was selected based on the following criteria: a) national population-based BCSPs or national evaluation reports of regional population-based screening programmes exist, and b) history (≥10 years) of implementation of their BCSP. Websites of the ministries of health or governmental offices in charge of the BCSPs were reviewed. The search results were shared with the M&E subgroup members with a request to submit any additional relevant documents that were not captured in the search. For each country, the most recently published documents were considered which either explicitly described recommended indicators for monitoring the BCSPs processes and outcomes or reported the results of performance indicator used. Finally, a list of definitions was compiled for those indicators which were identified in the eligible studies or originating from specific BCSPs’ documents.

### Development of a conceptual framework

Building on existing frameworks for making well-informed health care choices [15, 18, 19], a specific conceptual framework was adopted to guide the selection of potential performance indicators from those identified by the scoping review. The European QA Scheme served as the basis for the framework [15]. It describes several domains such as clinical effectiveness, safety, personal empowerment, and facilities and workforce that are intended to guide the quality evaluation of the breast cancer services. The European Observatory seminal document on assuring healthcare quality in the EU provided a number of other possible domains for our consideration such as equity, responsiveness and efficiency [19]. We also examined parameters of the Evidence to Decision framework that supports decision making in public health by assessing different options using explicit criteria [18].

### Selection of potential performance indicators

Selection of the final list of potential BCSP performance indicators was completed by means of two group exercises. First, all the identified candidates were grouped into indicator categories that generally represented certain steps along the breast cancer screening and diagnostic pathway (i.e., attendance, recall, screen-detected and interval breast cancer detection, sensitivity, mammographic quality, time requirements, biopsy, and treatment) (Additional file 1). During an in-person meeting of the M&E subgroup (Ispra, Italy, September 18, 2017), irrelevant and redundant indicators were removed from the initial list of performance indicators identified by the literature review. Irrelevant indicators were defined as those without sound clinical and/or empirical rationale, whereas indicators semantically very close to one another or those calculated in a very similar way were considered redundant. The decision to remove or retain an indicator was made by consensus among all subgroup members present at the meeting.

Second, a rating and ranking survey was created to assess the remaining performance indicators against pre-agreed criteria to facilitate the selection and make the process consistent. Table 1 presents the definition of the five criteria used in the survey (relevant, measurable, accurate, ethical and understandable). They were developed based on the criteria used for the selection of requirements for the European QA scheme [15], and the experience of other international organisations engaged in monitoring and evaluation activities in health care in general or in breast cancer specifically [20–23]. The M&E subgroup members discussed the criteria in light of their knowledge, analytical experience and data availability. Once the criteria were agreed upon, a weblink to complete the rating and ranking survey was sent to all the subgroup members via the SurveyMonkey platform (SurveyMonkey Inc., San Mateo, California, USA, www.surveymonkey.com). Participants were asked to rate each performance indicator identified by the literature searches based on the five criteria using a scale from 0 (completely disagree) to 10 (completely agree). For every indicator the average rating score and its standard deviation of each criterion were computed. Following the survey, the M&E subgroup of 20 members re-convened to review the responses in an in-person meeting and make the final selection.

**Table 1:** Selection criteria for the rating and ranking exercise

**RELEVANT** - An adequate indicator must have sound clinical and/or empirical rationale for its use. It represents an important aspect of breast cancer screening, gives useful information to different practice and policy stakeholders and stimulates efficient actions.	
**MEASURABLE** - The data required to assess the indicator must be available and easily accessible.	
**ACCURATE** - An adequate indicator should have a relatively large variation in the delivery of (sub)-processes of care to women between services and/or between Member States that is not due to random variation or female (client) characteristics.	
**ETHICAL** - Collection, treatment and analysis of indicator data respects individual rights of confidentiality, freedom of choice in providing data and informed consent about the nature and implications of data provided.	
**UNDERSTANDABLE** - An indicator has to be simple. Its interpretation should be easy and understandable by the majority of the population, not only by experts and stakeholders.	

Some breast cancer screening categories included more than one performance indicator; e.g. in the category “Attendance”, there were invitation coverage, participation rate, and screening coverage. In these cases, participants were asked to rank indicators within the category by appropriateness for inclusion on the final list of candidate BCSP performance indicators. A weighted average score was calculated for each ranked indicator. If there was only one indicator per category, participants were asked whether the indicator was appropriate for inclusion on the final list. For such indicators the proportion of positive and negative responses was calculated. As such, performance indicator selection was guided by the average rating score, weighted ranking score and/or the proportion of positive responses.

### BC screening and diagnostic pathway

**Fig. 1:**
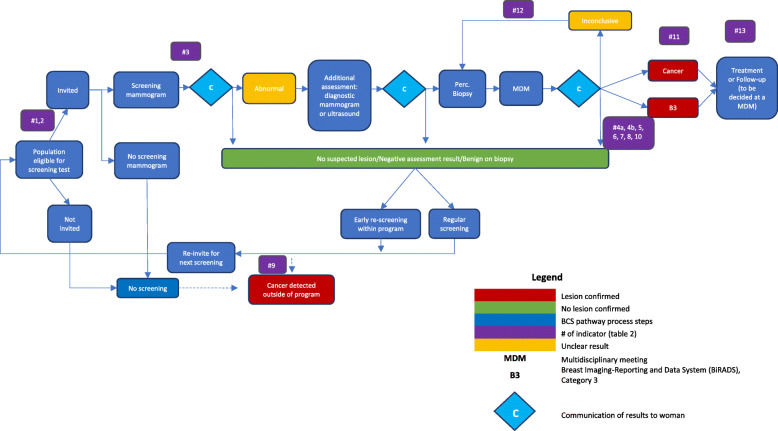
Breast cancer screening pathway

The final list of candidate indicators was mapped onto a breast cancer screening pathway that was developed by the M&E subgroup simultaneously to the indicator selection process (Fig. 1). This pathway illustrates the key steps of BCSP common to all EU countries and organises them in a logical order. The structure builds on the pathway presented by the European QA scheme and several other pathways published previously [15, 20, 24]. Of note, it was decided that the pathway for this exercise would predominantly cover BC screening, diagnosis and primary treatment steps.

## Results

### Scoping review

**Fig. 2:**
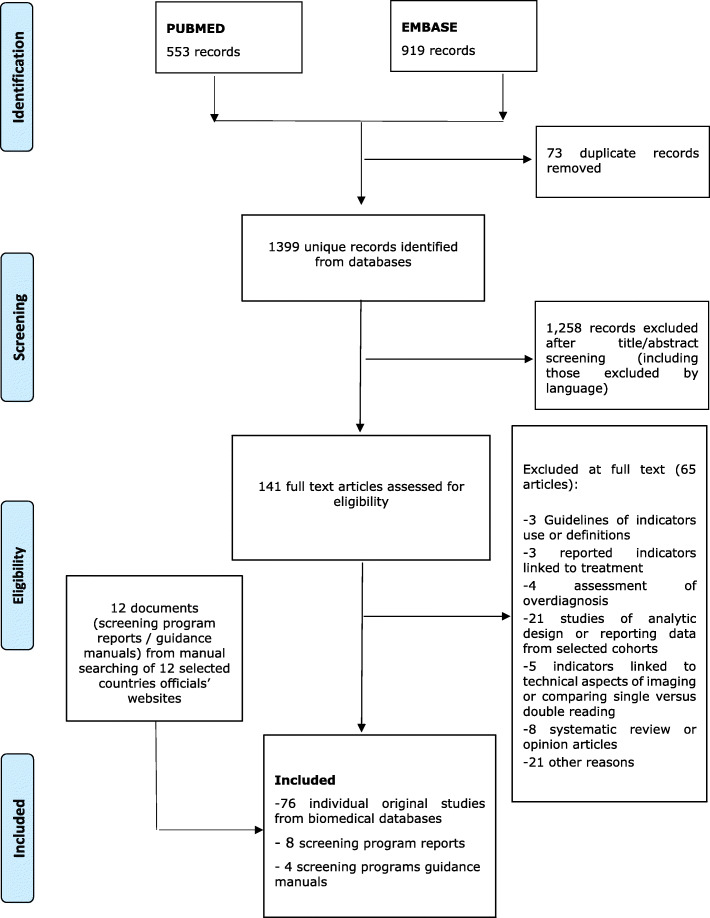
Flow chart for indicators selection from published articles

A total of 1399 unique citations were retrieved from the two databases (MEDLINE, EMBASE). 1258 citations were excluded based on title or abstract review. After reviewing the full texts of 141 citations, 76 studies were included for final review. Figure 2 presents the PRISMA flow chart for the selection process. All publications originated from the period 1994–2017 mainly from European Union countries, with the exception of three studies from Australia and four from Canada. The search of the grey literature yielded four BC screening guidance manuals (the European Union, Australia, Italy and England) and eight BCSP reports (Australia, Canada (2), Denmark, New Zealand, Scotland, Wales and the European Commission) which recommended or used process indicators for monitoring BCSP activities.

From the results of this published and grey literature review, an initial list of 1325 performance indicators was prepared. These indicators were reviewed by the panel of subgroup members to identify duplicates. A total of 96 unique indicators were finally retained.

### Performance indicators selection

Based on previous conceptual frameworks, the following domains to identify performance indicators for BCSPs were considered: clinical effectiveness, safety, facilities/resources/workforce, personal empowerment and experience, equity and cost-effectiveness. Clinical effectiveness was considered by the M&E subgroup as the most important domain, which is commonly supported by evidence (Additional file 1).

Out of the 96 identified indicators, 63 indicators were eliminated as irrelevant or redundant during the first in-person group exercise (Ispra, Italy, September 18, 2017) (Additional file 1). The subgroup modified the definition of three indicators (invitation coverage, interval cancer detection over expected ratio, false negative assessment after recall), added two new indicators (BC detection rate by subtype and time interval between screening and first treatment) that were not captured by the search, and one indicator (breast cancer detection rate) was split into two (one for initial and subsequent screenings). As a result, the group arrived at 39 indicators in total belonging to eight categories: attendance, recall, screen-detected and interval breast cancer, sensitivity, time requirements, biopsy, and primary treatment.

All 39 performance indicators (Additional file 1) were included into the online rating and ranking survey that was completed by the subgroup members (*n* = 20) between 6 and 15 November, 2017. The response rate was 65% (13 out of 20 experts), although only 11 (55%) respondents provided a complete response. Table 2 illustrates the results of the exercise using the example of recall. There were five indicator definitions in the recall category under review: recall rate, positive predictive value of recall, false positive rate, early recall rate, and false negative assessment after recall. By rating, the recall rate definition received the highest score across all 5 criteria, with the mean score ranging from 9.4 to 9.7. When ranking the five definitions, the recall rate definition was ranked #1 by 90% of the participants and had the highest weighted score. Full survey data on all 39 indicators is available upon request from the authors.

**Table 2:** Rating and ranking exercise results - the example of recall rate

**A. Definitions of indicators under review**					
**Name of indicators**	**Numerator**			**Denominator**	
**Recall rate**	n° of women recalled for further assessment based on a positive screening examination			n° of women screened	
**Positive predictive value of recall**	n° of breast cancers detected			n° of women recalled for further assessment	
**False positive rate**	n° of women recalled for further assessment with no cancer diagnosis			n° of women screened	
**Early recall rate**	n° of women invited to undergo a re-screen at an interval less than the routine screening interval			n° of women screened	
**False negative assessment after recall**	n° of women diagnosed with breast cancer after recall and negative further assessment			n° of women screened	
**B. Results**					
**Indicator selection criteria**	**Rating**^**a**^ **(mean, SD)**				
	**Early recall rate**	**Recall rate**	**False negative**	**False positive**	**Positive predictive value**
**Relevant**	7.5 (2.8)	9.4 (0.7)	8.3 (1.2)	9.1 (1.3)	9.0 (1.0)
**Measurable**	7.4 (2.5)	9.5 (0.9)	6.6 (2.5)	9.4 (0.7)	9.5 (0.8)
**Accurate**	6.9 (3.1)	9.4 (1.0)	6.9 (1.9)	9.4 (0.8)	9.0 (1.3)
**Ethical**	9.3 (0.8)	9.7 (0.5)	9.4 (0.7)	9.7 (0.5)	9.7 (0.5)
**Understandable**	8.7 (1.6)	9.4 (0.8)	7.9 (3.2)	9.0 (1.0)	9.3 (1.0)
**Rank**	**Ranking**				
**1**		9		2	
**2**	1	1		4	5
**3**	2		3	3	2
**4**	2				1
**5**	4				1
**Not applicable**	2		2	2	
**No of participants ranking**	11	10	11	11	9
**Weighted ranking score**	2.0	4.9	2.4	3.6	3.2

^a^ - a scale of 0 to 10 was used for rating

*SD* standard deviation;

Total number of participants was 13, 2 of whom provided only partial responses

Results were reviewed by the subgroup at the next meeting (Ispra, Italy, November 23, 2017) and 13 candidate performance indicators were finally selected. Those were: 1) screening coverage, 2) participation rate, 3) recall rate; 4) breast cancer detection rate, 5) invasive breast cancer detection rate; 6) cancers > 20 mm; 7) cancers ≤10 mm; 8) lymph node status; 9) interval cancer rate; 10) episode sensitivity; 11) time interval between screening and first treatment; 12) benign open surgical biopsy rate; and 13) mastectomy rate. Table 3 presents the final list of 13 performance indicators, their definitions and the domain of the conceptual framework they represent. The indicators were mapped on the BC screening pathway. Together, all 13 indicators cover several key steps along the pathway (Fig. 1) and address all the woman-important outcomes included in the new European guidelines Evidence to Decision framework, except overdiagnosis (Additional file 1) [25]. Additional file 1 shows the number of performance indicators that were identified at each key step of the selection process. Of note, this process and its results were presented to the entire GDG and QASDG.

**Table 3:** Final list of candidate performance indicators

Indicator	Definition	Conceptual framework domain	Indicator interpretation
**1. Screening coverage**	NUMERATOR: n° of women screened DENOMINATOR: n° of eligible (or target) women within a given period	Clinical effectiveness Facilities/resources/workforce Personal empowerment and experience	Measures the test coverage in the population. It should primarily be used for organised screening, but it can also include tests performed in the opportunistic setting. The aim is to maximise the value of the indicator, but it can only be applied to ages for which a strong recommendation for breast cancer screening has been given.
**2. Participation rate**	NUMERATOR: n° of women screened DENOMINATOR: n° of women invited	Clinical effectiveness Equity Personal empowerment and experience	The aim is to maximise the value of the indicator, but it can only be applied to ages for which a strong recommendation for breast cancer screening has been given.
**3. Recall rate**	NUMERATOR: n° of women undergoing further assessment for medical reasons based on a positive screening examination (either on the same day as screening or on recall) DENOMINATOR: n° of women screened	Clinical effectiveness Facilities/resources/workforce	Directly and timely measure the assessment workload and indirectly measure the false positive rates since cancers are a minority of recalls. High values indicate high false positive rates and should therefore raise concern.
**4. Breast cancer detection rate (4a: initial and 4b: subsequent screenings)**	NUMERATOR: n° of cancers screen-detected DENOMINATOR: n° of women screened	Clinical effectiveness	Indirect measure of screening sensitivity. Influenced by the underlying incidence and is higher in the prevalence (first) round. Geographical comparisons and trends should take into account these two determinants.
**5. Invasive breast cancer detection rate**	NUMERATOR: n° invasive screen-detected cancers DENOMINATOR: n° of women screened	Clinical effectiveness	Same as for the breast cancer detection rate.
**6. Cancers > 20 mm**	NUMERATOR: n° of invasive cancers > 20 mm screen-detected DENOMINATOR: n° of women screened	Clinical effectiveness	Diameter is a strong prognostic factor. Screening should act by reducing incidence of large cancers. A reduction in the proportion of large cancers is expected in women that have been already screened. Proportion during prevalence (first) round can be considered only to set a baseline, not to measure effectiveness.
**7. Cancers ≤ 10 mm**	NUMERATOR: n° of invasive cancers ≤10 mm screen-detected DENOMINATOR: n° of invasive cancers screen-detected	Clinical effectiveness	Indirect indicator of screening sensitivity. Reduction of the proportion of small screen-detected cancer among already screened women can be an early sign of loss in sensitivity. It is lower in the prevalence (first) round.
**8. Lymph node status**	NUMERATOR: n° of node-negative cancers screen-detected DENOMINATOR: n° invasive cancers screen-detected	Clinical effectiveness	Lymph node status is a strong prognostic factor. Screening showed efficacy in reducing the incidence of lymph node positive cancers. Furthermore, lymph node status influences the choice of treatment determining the use of chemotherapy or not in some cases.
**9. Interval cancer rate**	NUMERATOR: n° of interval cancers DENOMINATOR: n° of screened negative women at the last screening round	Clinical effectiveness	Direct measure of screening sensitivity. Influenced by the underlying incidence and the screening interval.
**10. Episode sensitivity**	NUMERATOR: n° screen-detected cancers DENOMINATOR: n° of all cancers detected	Clinical effectiveness	Direct measure of screening sensitivity. May be influenced by screening round, overestimating sensitivity during prevalence (first) round.
**11. Time interval between screening and first treatment**	Median number of days between screening and start of first treatment (10th percentile - 90th percentile)	Clinical effectiveness, Facilities/resources/workforce Equity	Measure the ability of the organisation to minimise the time required to identify, assess and treat cancers. Directly associated with women’s anxiety and, for extreme screening intervals. May reduce effectiveness because of cancer progression.
**12. Benign open surgical biopsy rate**	NUMERATOR: n° of women found not to have invasive cancer or DCIS after an open surgical biopsy DENOMINATOR: n° of women screened	Clinical effectiveness Safety	Direct measure of undesirable effects. Even if some of the benign lesions are treated because of their risk to progress to cancer.
**13. Mastectomy rate**	NUMERATOR: n° of women with mastectomy DENOMINATOR: n° of women screened	Clinical effectiveness Safety	Direct measure of the impact on treatment invasiveness. Identifying cancer at earlier stages should allow more conservative treatments.

## Discussion

In this paper, we have described the identification of candidate BCSPs performance indicators using a systematic process. There is a substantial overlap in BC screening processes selected for evaluation by our subgroup members and reports from other international BCSPs [20, 26]. Even if indicator definitions do not match precisely across various BCSPs, the programmes tend to focus on a small group of categories such as participation rate, cancer detection rates, including interval cancer rate, tumor size and time intervals. Further, the methodology for selecting performance indicators is consistent with previous research [27–29]. It consisted of iterative rating rounds of prioritisation with feedback given to the participants in face-to-face meetings. The addition of a ranking step was a novel modification. It allowed direct comparison and prioritisation of similar indicators within one indicator category. It also provided the subgroup with additional information to consider when making the final inclusion decision: a subset of indicators (e.g. proportion of tumours of various grade) was, for example, removed from further consideration because the respondents explicitly voted them non-applicable for monitoring and evaluation purposes.

Key strengths of this research are threefold. First, the set of performance indicators was identified using a systematic and methodologically rigorous approach. Second, the rating and ranking exercise proved helpful in facilitating indicator elimination. Third, the focused range of selected indicators can contribute to a better uptake of monitoring and evaluation activities across EU screening programmes.

We also note limitations. The response rate for the rating and ranking survey was acceptable but not as high as expected, although the vast majority of the participants provided a complete response. However, the purpose of the survey was to facilitate decision making. As such, the survey results were reviewed by all the M&E subgroup members at an in-person meeting that followed the survey. This allowed every member an opportunity to provide feedback, take part in the deliberations, and contribute to indicator selection. Further, despite the inclusion of a number of patient-important outcomes (e.g., breast cancer mortality, breast cancer incidence, quality of life, false positive), the list does not fully capture overdiagnosis and overtreatment. For the first, measuring overdiagnosis has been challenging even in trials [30] and large observational studies with long follow-up [31], thus finding operative measures for a timely monitoring seems conceptually impossible. For the latter, the indicator set covers invasiveness of treatment (i.e., mastectomy rate) and also has an indicator that is associated with the decision for chemotherapy (i.e. lymph node status).

## Conclusion

A systematic approach was employed to identify 13 BCSP candidate performance indicators. By documenting the process we facilitate its replicability on a wider scale. As such, this systematic and transparent process can be applied to developing indicators for other cancer and non-cancer programmes, as needed. However, this selection process should not be considered as complete without establishing the relationship between the indicators, aimed at measuring BCSP effectiveness, and breast cancer mortality. With the very limited evidence from randomised clinical trials as well as observational studies available [11–14], a methodology must be developed to measure these associations, as well as to determine, where possible, the acceptable and desirable levels of each of these performance indicators, or to determine whether benchmarking and trend monitoring are the only ways to interpret them. The methods and results of such assessment, which is ongoing (results expected in early 2021), will be described in another paper.

## Supplementary information

**Additional file 1: Appendix 1:** Search strategy. **Appendix 2:** Number of performance indicators identified per stage. **Appendix 3A:** Candidate indicators identified by a systematic review: pre-selected for the rating and ranking survey (*n* = 39). **Appendix 3B:** Candidate indicators identified by a systematic review: irrelevant and/or redundant (*n* = 63). **Appendix 4:** Conceptual framework considerations.

## Data Availability

The datasets used and/or analysed during the current study are available from the corresponding author on reasonable request.

## Abbreviations

BC: Breast cancer
BCSP: Breast cancer screening programme
ECIBC: European Commission Initiative on Breast Cancer
EU: European Union
GDG: Guidelines Development Group
JRC: Joint Research Centre
QA: Quality Assurance
M&E: Monitoring and Evaluation
UK: United Kingdom
