# Supplementary material for: Monitoring and evaluation of breast cancer screening programmes: selecting candidate performance indicators
Source: BMC Cancer. 2020 Aug 24;20:795. doi: 10.1186/s12885-020-07289-z (PMC7444070; doi:10.1186/s12885-020-07289-z)
Supplement: Supplementary file 1 — Additional file 1: Appendix 1: Search strategy. Appendix 2: Number of performance indicators identified per stage. Appendix 3A: Candidate indicators identified by a systematic review: pre-selected for the rating and ranking survey (n = 39). Appendix 3B: Candidate indicators identified by a systematic review: irrelevant and/or redundant (n = 63). Appendix 4: Conceptual framework considerations. [file 12885_2020_7289_MOESM1_ESM.docx]

**Appendix 1: Search strategy**

| **Search terms** | |
| --- | --- |
| **MEDLINE**  < OVID Medline Epub Ahead of Print, In-Process & Other Non-Indexed Citations, Ovid MEDLINE(R) Daily and Ovid MEDLINE(R) 1946 to Present>  02/03/2017 | 1     exp Breast Neoplasms/ (251008)  2     breast.ti. (227551)  3     1 or 2 (303764)  4     screen*.ti. (143961)  5     Early Detection of Cancer/ (15451)  6     4 or 5 (152151)  7     indicator*.ti,ab. (219966)  8     standard*.ti,ab. (1008563)  9     7 or 8 (1208924)  10     (evaluat* or impact* or performance or monitor*).ti,ab. (4329847)  11     3 and 6 and 9 and 10 (553) |
| **EMBASE**  Ovid <1974 to 2017 February 27>  02/03/2017 | 1     exp breast cancer/ (395810)  2     breast.ti. (295918)  3     1 or 2 (468914)  4     cancer screening/ (65839)  5     screen*.ti. (184632)  6     4 or 5 (222040)  7     indicator*.ti,ab. (277412)  8     standard*.ti,ab. (1386987)  9     7 or 8 (1636509)  10    (evaluat* or impact* or performance or monitor*).ti,ab. (5706083)  11     3 and 6 and 9 and 10 (1101)  12     11 not conference.so. (919) |

**Appendix 2: Number of performance indicators identified per stage**

| **Category of indicators** | **Number of indicators per stage** | | |
| --- | --- | --- | --- |
|  | **Systematic review** | **After exclusion of irrelevant and redundant indicators** | **After the rating and ranking exercise** |
| **Attendance** | 4 | 3 | 2 |
| **Recall** | 5 | 5 | 1 |
| **Breast cancer detection** | 34 | 23 | 5 |
| **Interval cancer** | 2 | 2 | 1 |
| **Sensitivity** |  | 2 | 1 |
| **Mammographic quality** | 7 | 0 | 0 |
| **Time requirements** | 9 | 2 | 1 |
| **Biopsy** | 27 | 1 | 1 |
| **Treatment** | 8 | 1 | 1 |
| **Total** | **96** | **39** | **13** |

**Appendix 3A: Candidate indicators identified by a systematic review: pre-selected for the rating and ranking survey (n=39)**

| **# of indicator** | **Category** | **Sub-category** | **Name of indicator** | **Numerator** | **Denominator** |
| --- | --- | --- | --- | --- | --- |
| 1 | **Attendance** |  | Attendance/participation rate | nº of women screened | nº of women invited |
| 2 | **Attendance** |  | Uptake rate/ coverage rate | nº of screened women | nº of eligible (or target) women within a given period |
| 3 | **Attendance** |  | Invitation coverage | nº of women invited | nº of eligible (or target) women within a given period |
| 4 | **Recall rate** |  | Recall/further assessment/referral rate | nº of women who were recalled for assessment (or as nº of further tests) | nº of women screened (or as nº of screen exams) |
| 5 | **Recall rate** |  | Early recall rate | nº of women invited to undergo a re-screen at an interval less than routine | nº of women screened |
| 6 | **Recall rate** |  | False negative assessment after recall | nº of women diagnosed with breast cancer after recall and negative further assessment | nº of women screened |
| 7 | **Breast cancer detection** | Detection rate | Breast cancer detection rate | nº of detected breast cancers | nº of women screened |
| 8 | **Breast cancer detection** | Detection rate | Breast cancer detection (over expected rate) | nº of breast cancers detected | nº of breast cancers expected (background expected incidence) |
| 9 | **Breast cancer detection** | Detection rate | Breast cancer detection over induction ratio | nº of breast cancers detected | nº of radiation induced breast cancers (derived from a linear-no-threshold risk model) |
| 10 | **Breast cancer detection** | Detection rate | False positive rate | nº of further assessments (including non-invasive and/or invasive procedures) with no cancer diagnosis | nº of screening mammograms (per 100 screening tests performed) |
| 11 | **Breast cancer detection** | Early stage cancer | Cancers stage I | nº of breast cancer stage I | nº of women screened |
| 12 | **Breast cancer detection** | DCIS | DCIS | nº of DCIS | nº of women screened |
| 13 | **Breast cancer detection** | DCIS | Proportion of DCIS cancers | nº of DCIS detected breast cancers | nº of breast cancers screen-detected |
| 14 | **Breast cancer detection** | Invasive cancer, low grade | Tumour grade % | nº of tumour grade | nº of invasive cancers detected |
| 15 | **Breast cancer detection** | Invasive cancer, low grade | Tumour grade I % | nº of tumour grade I | nº of invasive cancers detected |
| 16 | **Breast cancer detection** | High grade tumour | Tumour grade II % | nº of tumour grade II | nº of invasive cancers detected |
| 17 | **Breast cancer detection** | High grade tumour | tumour grade III % | nº of tumour grade III | nº of invasive cancers detected |
| 18 | **Breast cancer detection** | Advanced cancer | Cancers stage II+ | nº of breast cancer stage II+ | nº of women screened |
| 19 | **Breast cancer detection** | Advanced cancer | Cancers stage III+ | nº of breast cancer stage III+ | nº of women screened |
| 20 | **Breast cancer detection** | Advanced cancer | Proportional incidence of T2+ cancers over expected | nº of observed T2+ breast cancers | nº of expected T2+ breast cancers |
| 21 | **Breast cancer detection** | Invasive cancer | Invasive cancers | nº invasive screen-detected breast cancers | nº of women screened |
| 22 | **Breast cancer detection** | Invasive cancer | Invasive cancers ≤15 mm | nº of invasive breast cancers ≤15 mm | nº of women screened |
| 23 | **Breast cancer detection** | Invasive cancer | Invasive cancers ≤10 mm | nº of invasive breast cancers ≤10 mm | nº of women screened |
| 24 | **Breast cancer detection** | Invasive cancer | invasive cancers ≥11 mm | nº of invasive breast cancers ≥11 mm | nº of women screened |
| 25 | **Breast cancer detection** | Invasive cancer | Cancers >20 mm | nº of breast cancers >20 mm | nº of women screened |
| 26 | **Breast cancer detection** | Invasive cancer | Proportion of invasive cancers | nº of invasive breast cancers | nº of breast cancers screen-detected |
| 27 | **Breast cancer detection** | Invasive cancer | Invasive cancers ≤10 mm | nº of invasive breast cancers ≤10 mm | nº of invasive breast cancers screen-detected |
| 28 | **Breast cancer detection** | Invasive cancer | Invasive cancers ≤15 mm | nº of invasive breast cancers ≤15 mm | nº of invasive breast cancers screen-detected |
| 29 | **Breast cancer detection** | Lymph node status | Negative lymph node | nº of node-negative cancers | nº invasive cancers screen- detected |
| 30 | **Breast cancer detection** | Lymph node status | Negative lymph node | nº of node-negative cancers | nº of women operated |
| 31 | **Breast cancer detection** |  | Breast cancer detection rate by subtype | nº of women with cancer subtype | nº of women screened |
| 32 | **Breast cancer detection** | Positive predictive value | Positive predictive value (by test, by stage) | nº of breast cancers detected | nº of women recalled for further assessment |
| 33 | **Breast cancer detection** | Sensitivity | Episode sensitivity, detection method | nº breast cancer screen detected | nº of breast cancers detected |
| 34 | **Breast cancer detection** | Sensitivity | Sensitivity of the screening test | nº of screen-detected cancers | nº of screen-detected cancers + nº of post screen cancers detected 0 - <12 months |
| 35 | **Breast cancer detection** | Interval cancer rate | Interval cancer | nº of interval cancers | nº of screened women (also may be compared to expected breast cancer incidence rate) |
| 36 | **Breast cancer detection** | Interval cancer rate | Interval cancer detection over expected ratio | nº of observed interval cancers | Expected interval cancer incidence rate |
| 37 | **Time requirements** |  | Time interval between screening and the start of treatment | Number of days |  |
| 38 | **Biopsy** | Benign | Benign biopsy rate | nº of women found not to have invasive cancer or DICS after an open biopsy | nº of women screened |
| 39 | **Treatment** |  | Breast cancer treatment | nº of breast conservation surgeries (mastectomy, axillary dissection, no adjuvant therapy, radiotherapy only, chemotherapy only, hormonal therapy only combination therapy) | nº of women with a diagnosis of invasive breast cancer or DCIS |

| FNAC: fine needle aspiration cytology; NCB: needle core biopsy; VACB: vacuum assisted core needle biopsy; DCIS: ductal carcinoma in situ. |
| --- |

**Appendix 3B: Candidate Indicators identified by a systematic review: irrelevant and/or redundant (n=63)**

| **# of indicator** | **Category** | **Sub-category** | **Name of indicator** | **Numerator** | **Denominator** |
| --- | --- | --- | --- | --- | --- |
| **1** | **Attendance** |  | Cumulative examination rate | nº of participants in a given invitation round, who also participated in all previous rounds | nº of eligible women in a given invitation round, who were also eligible in all previous invitation rounds |
| **2** | **Attendance** |  | Retention rate | nº of screen-eligible women who had a subsequent screening mammogram within 30 months of a previous programme mammogram | nº of screen-eligible women with a programme mammogram in a given calendar year |
| **3** | **Recall rate** |  | Referral rate for invasive procedures for microcalcifications | nº of women referred for invasive procedures for microcalcifications | nº of women screened |
| **4** | **Recall rate** |  | Follow-up rate for microcalcifications | nº women who are recommended a one-year follow-up instead of the standard two-year screening interval); | nº of women screened |
| **5** | **Recall** **rate** |  | Eligible women re-invited within the specified screening interval | nº of women invited to screening within the optimal interval | nº of women invited to next screening round |
| **6** | **Breast cancer detection** | Detection rate | Microcalcification detection rate | nº of cancers detected in the presence of microcalcification | nº of women screened |
| **7** | **Breast cancer detection** | Non-invasive | Non-invasive/micro invasive cancers detected | nº of non-invasive cancers | nº of micro invasive cancers |
| **8** | **Breast cancer detection** | Advanced cancer | T2+ cancers, review errors | nº of T2+ cancers with previous mammogram reviewed and defined as lecture error | nº of T2 cancers detected |
| **9** | **Breast cancer detection** | Invasive cancer | Proportion of invasive screen-detected cancers <10 mm in size for which there was no frozen section | nº of invasive screen detected cancers <10 mm for which there was no frozen section | nº of invasive screen detected cancers <10 mm |
| **10** | **Breast cancer detection** | Interval cancer rate | Missed cancer rate | nº of missed cancers (not detected until a subsequent screening round) | nº of mammogram examinations |
| **11** | **Breast cancer detection** | Lymph node status | Sentinel node status | nº of patients where axillary status is determined with the sentinel node method | nº of patients where axillary status is assessed |
| **12** | **Breast cancer detection** | Sensitivity | Episode sensitivity, incidence method | one minus the incidence of interval breast cancers | expected incidence of breast cancer without screening |
| **13** | **Breast cancer detection** | Specificity | Specificity | nº of screened negative (true negative) | nº of true negative, plus the nº of false positive recalls as a result of abnormal mammograms, symptoms, and technical reasons (false positive) |
| **14** | **Breast cancer detection** | Interval cancer rate | Interval cancer, review errors | nº of interval cancers with a previous negative mammogram exam, subsequently reviewed and defined as an error | nº of interval cancers |
| **15** | **Mammographic quality** |  | Rate of diagnostic imaging | nº of abnormal screens referred by the screening radiologist that had either an ultrasound or special views performed in assessment | nº of abnormal screens referred by the screening radiologist |
| **16** | **Mammographic quality** | Acceptability of Mx | Proportion of women undergoing a technical repeat screening examination | nº of women with a repeat examination due to technical reasons | nº of women screened |
| **17** | **Mammographic quality** | Acceptability of Mx | Proportion of women with a radiographically acceptable screening examination | nº women with a radiographically acceptable screening examination | nº of women screened |
| **18** | **Mammographic quality** | Technical aspects | Radiation dose | mean glandular dose, which is the radiation dose measured on a 45 mm polymethylmethacrylate test phantom corresponding to a 53 mm standard breast. |  |
| **19** | **Mammographic quality** | Technical aspects | Target optical density | Logarithm (base 10) of the ratio between light intensity produced by a visible light source and perpendicularly incident on a film (Io), and light intensity transmitted by the film (I): OD = log10 (Io/I) |  |
| **20** | **Mammographic quality** | Technical aspects | Threshold contrast visibility | minimum detectable contrast for a 5-6 mm detail <1.5%. |  |
| **21** | **Mammographic quality** | Technical aspects | Spatial resolution | the smallest detectable detail at a defined contrast level to a given background. |  |
| **22** | **Time requirements** |  | Breast cancer diagnostic interval | nº of screen-eligible women with an abnormal screening mammogram result who were diagnosed within the recommended time interval (within 5 or 7 weeks of the abnormal mammogram result) | nº of screen-eligible women with an abnormal screening mammogram result in a given calendar year |
| **23** | **Time requirements** |  | Time between recall to assessment | nº of women assessed within 28 calendar days | nº of women recalled for assessment |
| **24** | **Time requirements** |  | Time between assessment and issuing of result | Measured as number of days or as proportion of patients that took ≤5 days |  |
| **25** | **Time requirements** |  | Time between screening mammography and issuing of result | Measured as number of days or as proportion of patients that took ≤15 days or ≤10 days |  |
| **26** | **Time requirements** |  | Time between result of diagnostic mammography and offered assessment | Measured as number of days or as proportion of patients that took ≤5 days |  |
| **27** | **Time requirements** |  | Time between result of screening mammography and offered assessment | Measured as number of days or as proportion of patients that took ≤5 days or ≤3 days |  |
| **28** | **Time requirements** |  | Time between symptomatic mammography and result | Measured as number of days or as proportion of patients that took ≤5 days |  |
| **29** | **Time requirements** |  | nº of assessment visits to obtain a definitive diagnosis | nº of women with ≤3 visits for diagnostic assessment and results appointments | nº of eligible women attending assessment |
| **30** | **Time requirements** |  | Receipt of screening results % (<2 weeks) | nº of adequately screened women sent results within 2 weeks | nº of screened women sent results |
| **31** | **Biopsy** | Benign | Benign open biopsy rate (in further assessed) | nº of women found not to have invasive cancer or DICS after an open biopsy | nº of women referred to assessment |
| **32** | **Biopsy** | Benign | Proportion of benign biopsy diagnosis on impalpable lesions weighing <30 grams | nº of benign biopsy diagnosis on lesions weighting <30 gm | nº of benign biopsy diagnosis on lesions weighting <30 gm |
| **33** | **Biopsy** | Benign biopsy | Specificity of FNAC | nº of correctly identified benign lesions | nº of benign lesions |
| **34** | **Biopsy** | Benign biopsy | Benign to malignant open biopsy ratio | nº of women undergoing open biopsy with benign result | nº of women undergoing open biopsy with malignant result |
| **35** | **Biopsy** | Benign biopsy group | Specificity of core biopsy | Nº of correctly identified benign lesions | nº of benign lesions |
| **36** | **Biopsy** | Insufficient results | Proportion of imaging guided FNAC procedures with insufficient result | nº of insufficient results | nº of FNAC procedures |
| **37** | **Biopsy** | Insufficient results | Proportion of FNAC/core biopsy at the diagnosis of cancer subsequently proven to be malignant with an insufficient result | nº of FNAC procedures with insufficient result with a subsequent malignant diagnosis | nº of FNAC procedures with insufficient result |
| **38** | **Biopsy** | Insufficient results | Proportion of image guided core/vacuum procedure with an insufficient result | nº of insufficient results | nº of image guided core/vacuum procedures |
| **39** | **Biopsy** | Open biopsy | Positive open surgical biopsy rate | nº of open surgical biopsies resulted in the diagnosis of cancer | nº screening mammographic examinations |
| **40** | **Biopsy** | Open biopsy | Open biopsy rate | nº of open surgical biopsies | nº of mammography screening examinations (also as nº referred for further assessment) |
| **41** | **Biopsy** | Preoperative biopsy | Proportion of patients subsequently proven to have breast cancer with a pre-operative FNAC or core biopsy at the diagnosis of cancer | nº of patients subsequently proven to have breast cancer | nº of patients having a pre-operative FNA or core biopsy at the diagnosis of cancer. |
| **42** | **Biopsy** | Preoperative biopsy | Proportion of patients subsequently proven to have clinically occult breast cancer with a pre-operative FNAC or core biopsy that is diagnostic for cancer | nº of patients subsequently proven to have clinically occult breast cancer. | nº of patients having a pre-operative FNA or core biopsy at the diagnosis of cancer. |
| **43** | **Biopsy** | Preoperative biopsy | Rate of fine-needle aspirations/core biopsy for diagnosis | Screens with a fine needle aspiration or core biopsy before any surgery in which the final result was primary malignant breast cancer | Screens with surgery in which the final result was primary malignant breast cancer |
| **44** | **Biopsy** |  | Accuracy | nº of cases with true benign and true malignant results | nº of all cases assessed by each diagnostic modality: FNA/ NCB/ VACB |
| **45** | **Biopsy** |  | Absolute sensitivity of core biopsy | nº of breast cancers diagnosed using the test | nº of breast cancers |
| **46** | **Biopsy** |  | Complete sensitivity of core biopsy | nº of breast cancers that were not reported as definitely negative or inadequate | nº of breast cancers |
| **47** | **Biopsy** |  | Absolute sensitivity of FNAC | nº of breast cancers diagnosed using the test | nº of breast cancers |
| **48** | **Biopsy** |  | Complete sensitivity of FNAC | nº of breast cancers that were not reported as definitely negative or inadequate | nº of breast cancers |
| **49** | **Biopsy** |  | False negative rate | nº of false negative diagnoses established by each needle biopsy modality | nº of cases with final malignant outcome |
| **50** | **Biopsy** |  | False positive (surgery) | nº false positive tests (from surgical or open biopsy) | nº of screened women (expressed as per 1000 participants) |
| **51** | **Biopsy** |  | Histological confirmation | nº of cancers histologically confirmed | nº of women screened |
| **52** | **Biopsy** |  | Image guided core biopsy malignant rate | nº of primary malignant breast cancers resulting from ultrasound- or stereotactic-guided core biopsies | nº of ultrasound- or stereotactic-guided core biopsies (all core biopsies included) |
| **53** | **Biopsy** |  | Impalpable lesions correctly identified at first open biopsy | nº of impalpable lesions correctly identified | nº of impalpable lesions |
| **54** | **Biopsy** |  | Inadequate specimen rate | nº inadequate specimens | nº of procedures assessed (FNA/ NCB/ VACB) |
| **55** | **Biopsy** |  | Modality for final diagnosis | the modality that ultimately established the diagnosis for each lesion they assessed | FNA/ NCB/ VACB /Calculated for all cores |
| **56** | **Biopsy** |  | Rate of stereotactic core biopsies / breast referred for microcalcifications | nº of stereotactic core biopsy procedures performed on a breast referred by the reading radiologist for microcalcifications in which the tissue was radiographed | nº of stereotactic core biopsy procedures performed on a breast referred by the reading radiologist for microcalcifications |
| **57** | **Treatment** |  | Proportion of localised impalpable lesions successfully excised during the first operation | nº localised impalpable lesions successfully excised during the first operation | nº of localised impalpable lesions |
| **58** | **Treatment** |  | Proportion of patients where a repeat operation is needed after incomplete excision | nº of reoperations due to incomplete excision | nº of operations for therapeutic purposes |
| **59** | **Treatment** |  | Proportion of wires placed within 1 cm of an impalpable lesion prior to excision | nº of wires placed within 1 cm of an impalpable lesion | nº of wires placed in impalpable lesions |
| **60** | **Treatment** |  | Breast conserving therapy | nº of women diagnosed with invasive cancer and treated with breast conserving therapy | nº of women operated on for invasive breast cancer |
| **61** | **Treatment** |  | Absence of re-operation due to postoperative complications | nº patients not requiring surgery | nº of patients with complications within 30 days after breast cancer surgery |
| **62** | **Treatment** |  | Staging of the axilla (patients who had axillary staging) | nº of women with invasive breast cancer with an axillary staging procedure | nº of women with invasive breast cancer |
| **63** | **Treatment** |  | Time between decision to operate and date offered for surgery | Measured as number of days or as proportion of patients that took ≤15 days or ≤10 days |  |

**Appendix 4: Conceptual framework considerations**

| **Domain of conceptual framework** | **Alignment with existing frameworks** | | | **Discussion** |
| --- | --- | --- | --- | --- |
|  | EU QA Scheme^[[1]](#footnote-1)^ | Evidence to Decision^[[2]](#footnote-2)^ | WHO EU observatory^[[3]](#footnote-3)^ |  |
| **Clinical effectiveness** | yes | yes  E.g.: How substantial are the desirable anticipated effects? What is the overall certainty of the evidence of effects? Is the problem a priority? | yes  E.g. Access to care; Timeliness | Outcomes are of special interest as they reflect the effectiveness of the programme. However, outcomes, especially long-term such as mortality, can be influenced by many factors outside of programme officers' control.  Therefore, in measuring programme performance it as important to collect information to measure processes (e.g., participate, time to assessment, etc) as it is through understanding and improving the processes that we can potentially influence the outcomes. |
| **Safety** | yes | yes  E.g.: How substantial are the undesirable anticipated effects? Do the desirable effects outweigh the undesirable effects? | yes  E.g.: Appropriateness | Harms associated with breast cancer screening that include the psychosocial burden of false positive results, overdiagnosis (i.e., tumors detected by screening that never would have led to clinical symptoms and needed treatment), as well as the associated discomfort caused to women, have been reported but are generally considered acceptable by women.   Although monitoring safety parameters may be seen by some as necessary, its operationalization is a challenge. Converting the identified harms into a M&E indicator is not always possible with harms such as psychological burden or discomfort due to their subjective nature. This type of data that typically requires administering surveys at regular intervals is not usually collected routinely and readily available. Even the extent of overdiagnosis, a seemingly more objective parameter, is not easily evaluated and is best assessed in a RCT with a long-term follow up^[[4]](#footnote-4)^^[[5]](#footnote-5)^. Calculating false-positive rates is probably the best available indicator alluding to the burden and is covered under CLE. |
| **Facilities, resources, workforce (FRW)** | yes | yes  E.g.: How large are the resource requirements? What is the certainty of the evidence of resource requirements?; Is the intervention feasible to implement? | yes  E.g.: Efficiency | FRW-related indicators deal with inputs to the BCS programme in terms of availability of appropriate facilities that conform to certain operational standards, resources available to run the facilities or the BCS programme, and properly trained staff qualified to perform mammograms, read the results, conduct biopsies, produce pathology reports, etc. |
| **Personal empowerment and experience** | yes | yes  E.g. Is there important uncertainty about or variability in how much people value the main outcomes? Is the intervention/option acceptable to key stakeholders? | yes  E.g.: Satisfaction; Acceptability; Continuity of care; Patient-centredness | Similar to Safety, these parameters do not lend themselves well to monitoring using routine data collection and indicators; Uptake and Retention rates may be the best among existing indicators that speak (rather indirectly) to acceptability and satisfaction: arguably, higher rates would imply greater satisfaction and acceptability of the programme. |
| **Equity** | no | yes  E.g.: What would be the impact on health equity? | yes  E.g.; Equity | Different from equality, equity implies considerations of fairness so that, in some circumstances, individuals will receive more care than others to reflect differences in their ability to benefit or in their particular needs. These differences often are influenced by socio-demographic factors such as race, income, education, etc.   A recent EU based study^[[6]](#footnote-6)^ has revealed issues with equity in access to BCS programmes among certain population groups: women without health insurance, women without residence permits, and women in prison. Access was determined by coverage and participation rates. However, inequities can be assessed in processes other than coverage and participation, e.g., communication and diagnostics time, as well as short-term (advanced stage disease, interim ca rate) and long-term outcomes (mortality). |
| **Cost-effectiveness (CE)** | no | yes  E.g.: Are the net benefits worth the incremental cost? | no | CE is an important factor in assessing performance of any screening programme. However, CE analysis cannot be converted into a routinely collected indicator for several reasons:  1) CE analysis requires modeling and implies a comparison with no screening or another screening modality: such data and skill may not be available to the programmes;  2) CE analysis evaluates a BCS programme as a whole, not its particular components/processes (e.g., participation rate, diagnostics interval), although the latter can be used for sensitivity analysis. |

1. EU Joint Research Center. European Quality Assurance scheme for Breast Cancer Services. *European Commission Initiative on Breast Cancer/Quality Assurance Scheme Development Group/Healthcare Quality Team* 2016; https://ecibc.jrc.ec.europa.eu/quality-assurance. [↑](#footnote-ref-1)
2. Alonso-Coello P, Schünemann HJ, Moberg J, et al. GRADE Evidence to Decision (EtD) frameworks: a systematic and transparent approach to making well informed healthcare choices. 1: Introduction. *BMJ (Clinical research ed).* 2016;353. [↑](#footnote-ref-2)
3. Legido-Quigley H, McKee M, Nolte E, Glinos AG. Assuring the quality of health care in the European Union: A case for action. Observatory Studies Series No.12: World Health Organization 2008. [↑](#footnote-ref-3)
4. Welch HG, Prorok PC, O’Malley AJ, Kramer BS. Breast-Cancer Tumor Size, Overdiagnosis, and Mammography Screening Effectiveness. *New England Journal of Medicine.* 2016;375(15):1438-1447. [↑](#footnote-ref-4)
5. Morris E, Feig SA, Drexler M, Lehman C. Implications of Overdiagnosis: Impact on Screening Mammography Practices. *Popul Health Manag.* 2015;18 Suppl 1(Suppl 1):S3-S11. [↑](#footnote-ref-5)
6. Deandrea S, Molina-Barcelo A, Uluturk A, et al. Presence, characteristics and equity of access to breast cancer screening programmes in 27 European countries in 2010 and 2014. Results from an international survey. *Preventive medicine.* 2016;91:250-263. [↑](#footnote-ref-6)
